# Comparative analysis of glass and Hemotek membrane feeding systems for malaria transmission research

**DOI:** 10.1093/trstmh/trac135

**Published:** 2023-01-13

**Authors:** Wouter Graumans, Michelle Schinkel, Geert-Jan van Gemert, Jeroen Spitzen, Teun Bousema, Pascal Miesen

**Affiliations:** Department of Medical Microbiology, Radboud University Medical Center, Radboud Institute for Health Sciences, Nijmegen 6500 HB, The Netherlands; Department of Medical Microbiology, Radboud University Medical Center, Radboud Institute for Molecular Life Sciences, Nijmegen 6500 HB, The Netherlands; Department of Medical Microbiology, Radboud University Medical Center, Radboud Institute for Health Sciences, Nijmegen 6500 HB, The Netherlands; Laboratory of Entomology, Wageningen University, Wageningen 6500 HB, The Netherlands; Department of Medical Microbiology, Radboud University Medical Center, Radboud Institute for Health Sciences, Nijmegen 6500 HB, The Netherlands; Department of Immunology and Infection, London School of Hygiene and Tropical Medicine, London WC1E 7HT, UK; Department of Medical Microbiology, Radboud University Medical Center, Radboud Institute for Molecular Life Sciences, Nijmegen 6500 HB, The Netherlands

**Keywords:** artificial feeding system, blood meal, gametocytes, *Plasmodium falciparum*

## Abstract

**Background:**

Glass membrane feeders are used in malaria research for artificial blood feeding. This study investigates the use of Hemotek membrane feeders as a standardized alternative feeding system.

**Methods:**

Hemotek feeders were compared with glass feeders by assessing mosquito feeding rate, imbibed blood meal volume and *Plasmodium falciparum* infection intensity on mosquito guts.

**Results:**

While mosquito feeding rate and blood meal volume were comparable between Hemotek and glass feeders, a loss in transmission was observed using the Hemotek feeder with a conventional collagen membrane. There was no difference in transmission between both feeders when Parafilm was used as the membrane.

**Conclusions:**

Hemotek feeders with a Parafilm membrane can be used as an alternative feeding system for malaria transmission research.

## Introduction

Malaria remains a major public health problem, with 247 million cases and 619 000 deaths in 2021.^1^ Parasite transmission can occur when *Anopheline* mosquitoes imbibe sexual-stage parasites (gametocytes) that are present in the blood of malaria-infected individuals. Membrane feeding assays (MFAs) allow artificial blood feeding of mosquitoes and are fundamental tools in malaria transmission research. Membrane feeding is used for mosquito colony maintenance and for research purposes in the form of direct and standard membrane feeding assays (DMFAs and SMFAs, respectively). DMFAs are commonly used to evaluate the infectiousness of naturally infected gametocyte carriers while SMFAs are the gold standard assays to quantify the capacity of immune factors or compounds to prevent transmission of *in vitro* cultured gametocytes. Malaria MFAs generally use water-jacketed membrane feeders from handcrafted glass that are connected to a circulating warm water bath and covered with stretched Parafilm as a feeding membrane. The Hemotek feeding system is a viable alternative that uses electrical heating of metal feeder reservoirs and the blood is covered by a collagen or stretched Parafilm membrane for insects to feed from. Hemotek feeders are commonly used in *Aedes* research for colony maintenance and infection experiments with arboviruses,^2^ but their current use is limited in malaria research and sometimes Hemotek systems have been abandoned in favour of glass feeders after observing low *Plasmodium falciparum* infection rates.^3^ Compared with custom-made glass feeders, commercial Hemotek feeders have advantages in terms of standardization, temperature control and use as stand-alone systems with a single feeder. However, the Hemotek system has never been validated directly against glass feeders. We therefore compared both feeding systems and assessed the feeding rate of *Anopheles stephensi*, imbibed blood meal volume and performance in *P. falciparum* transmission experiments.

## Methods

*An. stephensi* mosquitoes (Sind-Kasur strain) were maintained at 30°C and 80% humidity with a reverse day–night cycle (12:12). *P. falciparum* parasites (strain NF135) were cultured in an automated incubator. Venous blood was collected in LiHep BD Vacutainer tubes (#368496; Sanquin, Nijmegen, The Netherlands). Cages containing 50 mosquitoes were exposed for 10 min to feed as described previously,^4^ using glass midi-feeders or the Hemotek feeding system (PS6120) with 1 ml reservoirs (FU1-1). Parafilm (M laboratory film) or Hemotek collagen (MEM5) were used as feeding membranes. The blood feeding rate was first assessed by visual inspection and unfed and partially fed mosquitoes were removed. For fully engorged mosquitoes, the blood meal volume was measured in dissected abdomens immediately after feeding using the Hemoglobin Colorimetric Assay Kit (#700540; Cayman Chemical, Ann Arbor, MI, USA) as described previously.^4^ Mosquito infectivity was assessed 6–8 d post-infection in a sample of 20 mosquitoes by counting the number of established oocyst in dissected 1% mercurochrome-stained midguts. Linear and negative binomial mixed effects models were used to compare feeding performance and oocyst densities between conditions, accounting for the relatedness of observations from the same experiments.

## Results and Discussion

**Figure 1.**
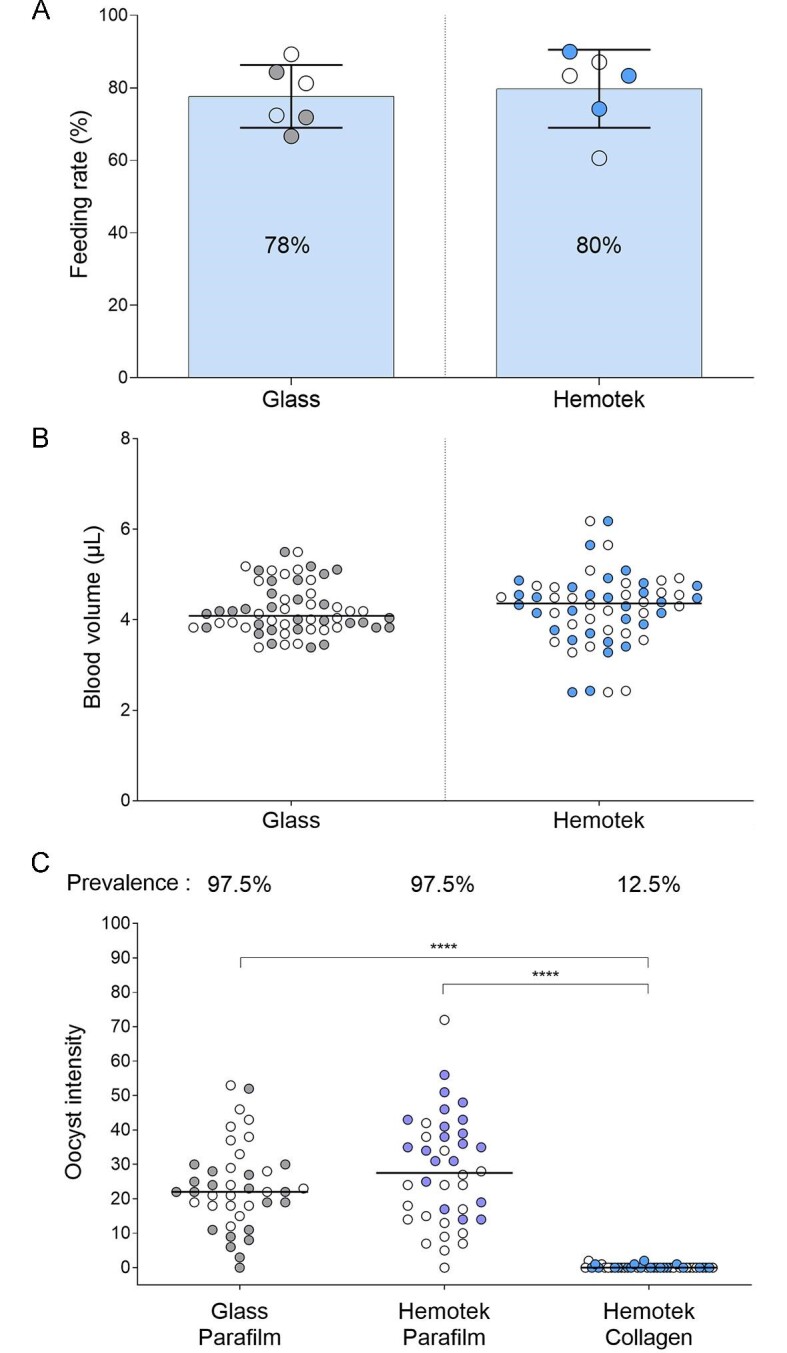
Feeding performance and *P. falciparum* transmission intensity comparing glass against Hemotek membrane feeders. To compare mosquito membrane feeding rates between glass feeders (Parafilm membrane) and Hemotek feeders (collagen membrane), six cups containing 50 mosquitoes were prepared for each setup. Blood feeding was scored by eye and reported as a percentage of the total mosquitoes fed. **(A)** For glass feeders, the mean feeding rate was 77.64% (standard deviation [SD] 8.66, range 66.67–89.29) and for Hemotek feeders it was 79.76% (SD 10.79, range 60.61–90). Subsequently, 20 fully engorged blood-fed mosquitoes from each cup were selected to assess the imbibed blood meal volume. **(B)** Blood meal volume was not significantly different between glass and Hemotek feeders (p=0.83), with a mean volume of 4.23 µl (SD 0.56, range 3.39–5.5) and 4.26 µl (SD 0.79, range 2.4–6.18), respectively. Transmission was assessed in an MFA using cultured *P. falciparum* gametocytes. In duplicate cups per condition, 50 mosquitoes were fed on glass membrane feeders with a Parafilm or Hemotek feeders with a collagen or Parafilm membrane for 10 min. Infection intensity was measured by the number of developed oocysts 6–8 d post-infection on the mosquito midgut in 20 dissected mosquitoes per feeder. Oocyst densities were significantly lower in Hemotek feeders with a collagen membrane when compared with both Hemotek with Parafilm and glass feeders with Parafilm (p<0.0001). **(C)** There was no difference in oocyst density between Hemotek and glass feeders when Parafilm was used as the membrane (p=0.44). Duplicate experiments are shown pooled (open and closed circles). ****p≤0.0001.

When comparing Hemotek feeders with a collagen membrane against our standard laboratory set-up with glass feeders and Parafilm, we observed no statistically significant difference in mosquito feeding rate (mean of 80% and 78%, respectively; p=0.94) or imbibed blood meal volume (median 4.36 µl and 4.09 µl, respectively; p=0.83) (Fig. 1A, B). Upon feeding on cultured *P. falciparum* gametocytes, oocyst densities were significantly lower in Hemotek feeders when using the Hemotek collagen membrane (p<0.0001) (Fig. 1C). This reduction in transmission efficiency with the collagen membrane may be explained by several factors, including temperature stability, binding of gametocytes by electrostatic forces and the selected feeding membrane. Since the type of feeding membrane has previously been reported as a source of variation in mosquito feeding rate and blood volume,^5,6^ we first tested Parafilm as a membrane on Hemotek feeders. For Hemotek feeders we observed a marked improvement in infection rates for Parafilm compared with collagen membranes (p<0.001). There was no statistically significant difference in oocyst density between Hemotek and glass feeders when parafilm was used as the membrane (p=0.44) (Fig. 1C).

These findings support the use of Hemotek feeders with Parafilm for malaria transmission research. Hemotek feeders are advantageous for small-scale experiments where only a few feeders are needed at the same time. Hemotek feeders are commercially available, do not need specialized glass craftmanship—thus removing a potential source of variation—and are less fragile. However, in an experimental setup where a large number of feeders are used concurrently, glass feeders may be preferred since they can be connected in a single chain to the same circulating system, which is more cost effective than multiple Hemotek setups.

### Conclusions

We investigated the use of Hemotek membrane feeders as a standardized alternative feeding system for feeding *P. falciparum* gametocytes to *An. stephensi* mosquitoes. We conclude that Hemotek feeders with a Parafilm membrane (but not a collagen membrane) can be used as an alternative feeding system for malaria transmission research.

## Data Availability

The datasets used and/or analysed during the present study are available from the corresponding author upon request.
